# How much can music listening habits tell us about mental health? An interpretable machine learning benchmark using genre preference and self-reported anxiety, depression, insomnia, and obsessive–compulsive symptoms

**DOI:** 10.3389/fpsyg.2026.1970053

**Published:** 2026-10-26

**Authors:** Yakun Li, Binxin Ren

**Affiliations:** 1 School of Music, Sichuan Normal University, Chengdu, Sichuan, China; 2 Zhonghe High School Chengdu Sichuan, Chengdu, Sichuan, China

**Keywords:** class imbalance, convenience sample, interpretable machine learning, mental health, music preference, predictive modelling, self-report, SHAP

## Abstract

**Background:**

Music listening is widely believed to index mental health, and this belief underwrites applications inferring psychological state from listening records. We estimated how much information musical preference and listening behavior carry about self-reported distress, and tested whether classification accuracy reported for a widely reused public dataset reflects discriminative skill or class imbalance.

**Methods:**

Cross-sectional secondary analysis of the Music and Mental Health (MxMH) survey (*n* = 736), a self-selected online convenience sample collected in 2022 with no probability sampling frame, no design weights, and no measurement of gender, geography, education, income, or clinical history. In total, 42 predictors covering genre exposure, favorite genre, streaming platform, listening hours, tempo preference, and musical engagement were modeled against four single-item, unvalidated self-anchored distress ratings, a secondary composite index, and a self-attributed judgment of whether music improves mental health. Elastic net, support vector machine, random forest, and gradient boosting models were compared against mean-prediction and majority-class baselines using nested cross-validation, corrected *t*-tests, permutation nulls, and Shapley additive explanations.

**Results:**

Out-of-sample explanatory power was small. Random forests achieved cross-validated *R*^2^ values of 0.068 for depression, 0.058 for the composite index, 0.032 for anxiety, and 0.006 or below for insomnia and obsessive–compulsive ratings, exceeding the mean-prediction baseline for all outcomes except insomnia (*p* = 0.157). Permutation tests separated depression, anxiety, the composite, and obsessive–compulsive ratings from noise, but not insomnia (*p* = 0.198). For the self-attributed effect of music, the best classifier reached 74.7% accuracy against a majority-class baseline of 74.5% (*p* = 0.896), while balanced accuracy peaked at 0.551 and the Matthews correlation coefficient at 0.135.

**Conclusion:**

Listening habits carry a real but very small signal about self-reported distress, and apparent accuracy on this dataset is an artifact of class imbalance. Musical preference is not a usable individual-level marker of psychological state. These estimates come from a single, heavily reused, non-representative convenience sample with unvalidated single-item outcomes and no demographic weighting variables, so they characterize this archive rather than any defined population and require independent replication before any specific effect size is treated as settled. Dataset: MxMH survey, Kaggle.

## 1 Introduction

The belief that music benefits mental health is widely held. Listeners report selecting particular music when anxious, sleepless or low, and describe those choices as deliberate acts of self-management. Research gives the belief partial support. Systematic reviews report modest benefits of music-based interventions for wellbeing (Viola et al., 2023), for anxiety in controlled listening studies (Harney et al., 2023) and for sleep quality among adults with mental health problems (Zhao et al., 2024). Adolescents and young adults, the group that listens most, are also the group for whom the evidence base is thinnest (McFerran et al., 2020).

A second claim has grown alongside the first, and it is a claim of a different kind. If musical behavior reflects psychological state, then musical behavior should be readable. Streaming platforms hold detailed records of what people listen to, when, and for how long, and applications increasingly promise to infer mood from that data. The implicit premise is that taste and listening habits form a signal dense enough to support individual-level prediction. That premise is empirical and can be tested directly.

Theory suggests the signal may be weak. Music listening is understood as strategic affect regulation, serving self-awareness, social relatedness, and arousal regulation simultaneously (Schäfer et al., 2013), so the same regulatory goal can be pursued through very different musical means. An important refinement distinguishes adaptive from maladaptive engagement (Saarikallio et al., 2015). Studies in this tradition repeatedly find that the maladaptive dimension, rather than genre preference, mediates the relationship with symptom severity: in a large Indian sample, maladaptive engagement correlated with the total depression score (*r* = 0.39), whereas adaptive engagement was instead related to trait positive affect (Kanagala et al., 2021). Borderline personality symptoms likewise relate indirectly to preference, through the functions music serves (Kowalewska et al., 2024). Preference itself is reliably structured and linked to cognitive style (Greenberg et al., 2015), but replication finds weaker trait associations than early reports suggested (Chee et al., 2024), and preferences shift with context, as studies conducted during the coronavirus disease 2019 (COVID-19) pandemic have shown (Krause et al., 2025; Ziv and Hollander-Shabtai, 2022; Hennessy et al., 2021; Vidas et al., 2021; Ferreri et al., 2021). If the psychologically active variable is how a person uses music rather than what they play, a dataset that measures genre exposure in detail but function not at all should predict poorly.

Testing that premise rigorously presents several difficulties. Predictive claims in psychology and psychiatry shrink under scrutiny: reported classification accuracy in small neuroimaging studies of depression falls systematically as sample size grows, the opposite of what a genuine signal should do (Flint et al., 2021). Validation procedures that pool information across training and testing partitions produce strongly biased estimates at sample sizes far larger than those typical in music psychology (Vabalas et al., 2019). Comparisons between algorithms compound the difficulty, because repeated cross-validation folds share training data and the ordinary paired *t*-test understates the variance of the score difference (Nadeau and Bengio, 2003). A further hazard is that *post-hoc* explanation of a model with poor discrimination produces confident narratives about associations the model has not learned (Petch et al., 2022). These problems are not confined to music psychology. A wide range of low-burden behavioral and physiological signals, from smartphone mobility, phone use and sleep to vocal characteristics, is now catalogued as a basis for inferring psychiatric state (Bufano et al., 2023), and where one of those signals has been reviewed against explicit methodological criteria the picture is not reassuring: a scoping review of voice-based screening for depression and anxiety reported a median total sample of 118 participants, external validation absent in 68% of evaluations, only 11% of evaluations rated as having high clinical readiness, and accuracy commonly reported without the class distribution needed to interpret it (Muntean-Codrea et al., 2026). A study in that field has also shown that measured performance depends heavily on how the behavioral sample is collected rather than on model architecture alone (Vilenchik et al., 2025). A strict benchmark of an intuitive behavioral signal therefore speaks to a problem shared well beyond the present dataset.

This study applies the validation protocol implied by these lessons to a widely reused public dataset (Rasgaitis, 2022). Its categorical outcome is severely imbalanced, with roughly three-quarters of respondents endorsing a single option, so any accuracy figure for that outcome must be read against the base rate before it can be interpreted. Two questions are addressed. First, how much variance in self-reported distress can musical preference and listening behavior explain out of sample, once model selection is nested inside cross-validation and performance is referenced against trivial baselines and permutation nulls. Second, whether an apparently strong accuracy figure on the categorical outcome reflects discriminative skill or the base rate. Five hypotheses were tested. Hypothesis 1: Listening behavior will carry more predictive information than genre preference. Hypothesis 2: Out-of-sample explanatory power will be small, with cross-validated *R*^2^ below 0.15, and non-linear ensembles will not substantially outperform regularized linear models. Hypothesis 3: Age will be among the strongest predictors, and apparent genre effects will attenuate once age is included. Hypothesis 4: Apparent classification accuracy will exceed 70% while balanced accuracy and the Matthews correlation coefficient remain near chance. Hypothesis 5: At least one association will be non-monotonic across its range.

## 2 Materials and methods

### 2.1 Data source and participants

Data come from the Music and Mental Health (MxMH) survey, a publicly archived online questionnaire distributed through social media and community forums and made available through Kaggle (Kaggle, Inc., San Francisco, CA, USA) (Rasgaitis, 2022). The archived file contains 736 complete submissions and 33 variables, with submission timestamps from 27 August 2022 to 9 November 2022. Recruitment was open and self-selected. No probability sampling frame was used, no quotas were applied, and no design weights exist, so the sample is a non-probability convenience sample and no claim of representativeness is made. The instrument included a consent acknowledgment item; all 736 records carry the same value, so the field is a constant and was removed before modeling.

Respondents ranged in age from 10 to 89 years (mean: 25.21, SD: 12.05, median: 21). A total of 153 respondents (20.8%) were under 18 years, and 27 were aged 60 or above, so the sample is dominated by adolescents and young adults. Gender, geographic location, education, income, ethnicity and clinical history were not collected, which prevents both demographic weighting and adjustment for the confounders usual in this literature.

### 2.2 Measures

Four outcomes were recorded as single self-anchored ratings from 0 to 10, labeled anxiety, depression, insomnia and obsessive–compulsive disorder (OCD). No validated instrument was administered, and there is no clinical corroboration or diagnostic threshold. This is most acute for the obsessive–compulsive item, where colloquial usage diverges from the diagnostic construct. We therefore refer to self-reported distress rather than to symptoms or disorders. Descriptive statistics for all variables appear in Table 1.

**Table 1:** Descriptive statistics for the analytic sample (*n* = 736).

Continuous variable	*n*	Mean	SD	Minimum	Median	Maximum
Age (years)	735	25.21	12.05	10.0	21.0	89.0
Daily listening (h)	731	3.45	2.62	0.0	3.0	16.0
Tempo of preferred genre (BPM)	621	123.76	32.03	40.0	120.0	220.0
Genre breadth (0–16)	736	5.93	2.61	0.0	6.0	16.0
Genre exposure intensity (0–3)	736	1.16	0.41	0.2	1.1	3.0
Anxiety (0–10)	736	5.84	2.79	0.0	6.0	10.0
Depression (0–10)	736	4.80	3.03	0.0	5.0	10.0
Insomnia (0–10)	736	3.74	3.09	0.0	3.0	10.0
Obsessive–compulsive rating (0–10)	736	2.64	2.84	0.0	2.0	10.0

Categorical variable	*n*	%
Self-reported effect of music: improve	542	73.6
Self-reported effect of music: no effect	169	23.0
Self-reported effect of music: worsen	17	2.3
Listens while working (yes)	579	78.7
Plays an instrument (yes)	235	31.9
Composes music (yes)	126	17.1
Explores new music (yes)	525	71.3
Foreign-language music (yes)	404	54.9
Favorite genre: rock	188	25.5
Favorite genre: pop	114	15.5
Favorite genre: metal	88	12.0
Favorite genre: classical	53	7.2
Favorite genre: video game music	44	6.0

Tempo values outside a 40–220 BPM window and daily listening values above 16 h were set to missing before these statistics were computed. Genre exposure intensity is reported for description only and was excluded from all models, as it is a perfect linear combination of the 16 genre exposure items. Percentages for categorical variables are of the full sample of 736. Full genre exposure frequencies, the correlation matrix, and variance inflation factors are provided in the Supplementary material. SD, standard deviation; BPM, beats per minute.

A composite distress index was formed as the mean of the four standardized items. Internal consistency was modest (Cronbach alpha: 0.659, McDonald omega: 0.670), with single-factor loadings of 0.73 for anxiety, 0.70 for depression, 0.47 for insomnia, and 0.40 for obsessive–compulsive ratings. Two of these four loadings are weak, so the composite is not treated as a primary outcome parallel to depression. It is retained as a secondary, descriptive summary of general distress for two specific reasons: it is the only outcome in this dataset for which an internal consistency coefficient is estimable at all, which makes it the only outcome whose measurement-error ceiling can be computed rather than assumed, and averaging four noisy items provides a check on whether the very low single-item results are an artifact of item-level unreliability. Results for the four individual items are reported alongside it throughout and carry the substantive conclusions. The modest reliability of these measures caps the variance any predictor set can explain, a point quantified in Section 4.

The survey included 16 genre exposure items asking how often the respondent listens to classical, country, electronic dance music (EDM), folk, gospel, hip hop, jazz, K-pop, Latin, lo-fi, metal, pop, rhythm and blues (R&B), rap, rock and video game music, with four ordered options from never to very frequently, scored 0 to 3. A separate item recorded the single favorite genre. Primary streaming service was recorded in six categories. Daily listening hours and the tempo of the preferred genre, in beats per minute, were entered as open numeric responses. Five binary items captured musical engagement: listening while working, playing an instrument, composing, actively exploring new material, and listening to music in unfamiliar languages. A final item asked whether music improves, does not affect, or worsens the respondents’ mental health; this is a subjective attribution rather than an observed change. Genre breadth was derived as the count of genres rated at least sometimes.

### 2.3 Data preparation

Screening rules were fixed before modeling. The archived tempo variable contains values that cannot correspond to a musical tempo, including a maximum of 999,999,999 and a further value of 624, so a plausibility window of 40 to 220 beats per minute was imposed; 2 values fell above and 6 below, reducing 629 non-missing values to 621 (median 120). Daily listening hours contained 5 values above 16 h, including one report of 24 h; these were set to missing, leaving 731 valid observations. No respondent was deleted, only affected cells. Favorite genre categories with fewer than 20 respondents (gospel, Latin, and lo-fi) were combined into a residual category, and the Pandora streaming category was merged with other services because, with the full sparse indicator set, maximum likelihood estimation of the logistic model failed due to a singular Hessian.

The final design matrix contained 42 predictors: age, daily listening hours, tempo, 5 binary engagement indicators, 16 ordinal genre exposure items, genre breadth, 12 favorite-genre indicators, and 4 streaming-service indicators. Variance inflation factors reached a maximum of 7.76 for genre breadth, with 2 values above 5 and none above 10. Genre exposure intensity, the mean of the 16 ordinal items, is a perfect linear combination of them and was excluded from all models.

Missingness was concentrated in tempo, missing for 115 respondents (15.63%); all other variables were missing for 8 respondents or fewer. The test performed by Little (1988) rejected the hypothesis that values were missing completely at random, *χ*^2^(22) = 41.03, *p* = 0.008. Respondents with missing tempo were older (mean 28.42 vs. 24.62 years, *t* = 2.64, *p* = 0.009) and reported fewer daily listening hours (mean 2.62 vs. 3.60 h, *t* = 4.72, *p* < 0.001), but missingness was unrelated to any outcome (*p* = 0.150–0.964) and to favorite genre, *χ*^2^(15) = 22.49, *p* = 0.096. These results support the missing-at-random assumption conditional on observed covariates. Multiple imputation by chained equations with five imputations was used for the parametric models (van Buuren and Groothuis-Oudshoorn, 2011), and the imputed datasets were pooled according to Rubin’s (1987) rules. Within the machine learning pipelines, median imputation was fitted inside each training fold and applied to the corresponding validation fold, so no information crossed the partition boundary.

### 2.4 Parametric baseline models

Ordinary least squares models were estimated for each outcome using heteroskedasticity-consistent 3 (HC3) standard errors, entering all 42 predictors simultaneously. The model equation and the definitions of its terms are given in the Supplementary material. Quantile regressions at the 25th, 50th, and 75th percentiles are also reported there.

For the self-attributed effect of music, a binary logistic model contrasting improvement with all other responses was estimated, along with its specification, and is provided in the Supplementary material.

Coefficients from that model are reported as odds ratios (ORs). A multinomial specification retaining all three categories was estimated using a reduced set of predictors because the smallest category contained only 17 respondents.

### 2.5 Machine learning models and validation

Four algorithm families were compared: elastic net regularized linear models, support vector machines with a radial basis kernel, random forests, and gradient boosted trees. Before any learning algorithm was evaluated, a mean-prediction regressor and a majority-class classifier were fitted under the identical scheme; their scores appear in every performance table. Performance was estimated using nested cross-validation with an outer scheme of 5 folds repeated twice, yielding 10 outer estimates, and an inner 3-fold randomized search over 8 hyperparameter configurations. Single hold-out splits were not used, because at *n* = 736 they are too unstable to support these comparisons (Vabalas et al., 2019). The full procedure appears in Algorithm 1, Figure 1. *The regularized objective, minimized by the gradient-boosted trees, and the definitions of its terms are given in the* *Supplementary material*.

ALGORITHM 1 **Nested cross-validation with leakage control and gated interpretation.**

Input: design matrix *X* (*n* = 736, *p* = 42), outcome *y*, model families *M*, grid *G*Output: unbiased performance estimate, corrected comparisons, gated attributions 1   for each outer repeat *r* = 1, 2 do 2       partition the sample into 5 folds 3       for each outer fold *k* = 1 … 5 do 4           Xtrain, ytrain ← all folds except *k* 5              Xtest, ytest ← fold *k* 6           fit imputer and scaler on Xtrain only # leakage control 7           for each model family *m* in *M* do 8               inner 3-fold randomized search over *G*(*m*) 9               refit m on all of Xtrain with selected hyperparameters 10              score m on Xtest, store the fold result 11  compare families with the corrected *t* statistic of Equation 3 12  refit the winning family on the full sample 13  for *b* = 1 … *B* do # permutation null; *B* = 100 regression, 200 classification 14      shuffle *y*, repeat 5-fold cross-validation, store the score 15  if observed score ≤ 97.5th percentile of the null then 16      report the outcome as not distinguishable from chance, stop 17  else 18      compute SHAP values and permutation importance (Equation 4)

**Figure 1:**
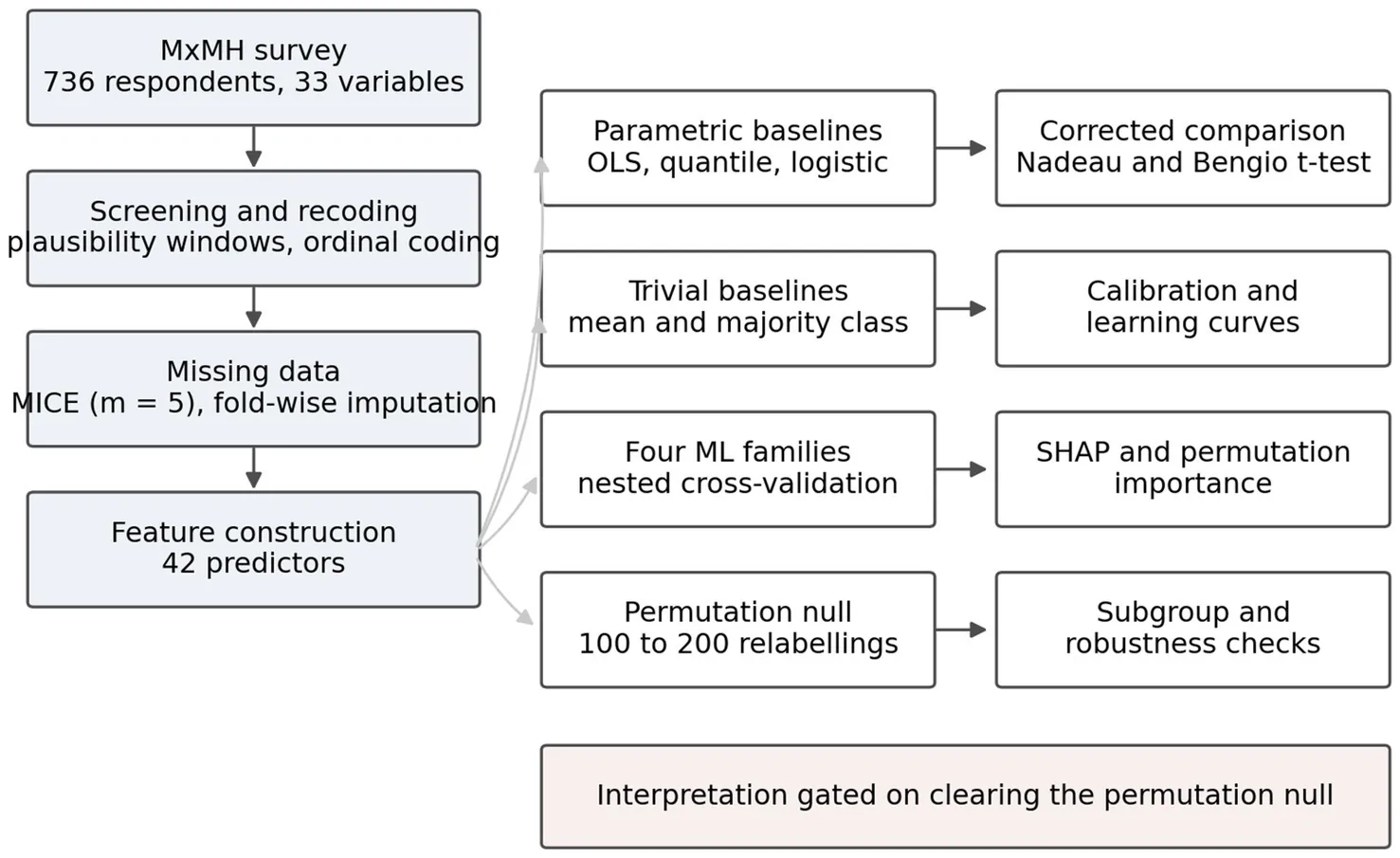
Analytical pipeline from the archived survey file to conditionally gated interpretation. Data preparation (left column) proceeds through screening, imputation, and feature construction. Model estimation (center column) places parametric and trivial baselines alongside four machine learning families and a permutation null. Evaluation (right column) applies corrected comparison, calibration, learning curves, feature attribution and robustness analysis. *Post-hoc* explanation is applied only after a model clears its permutation null, as specified at lines 15–18 of Algorithm 1.

### 2.6 Class imbalance and evaluation metrics

The self-attributed effect variable is severely imbalanced (improve 542, no effect 169, worsen 17). Three specifications were estimated: the three-category outcome as collected, a binary contrast of improvement against all other responses, and an assessment of whether the worsening category can be modeled at all. Class weighting was the primary approach to imbalance. Because accuracy is uninformative under this degree of imbalance, two imbalance-sensitive metrics carry the argument and are defined explicitly. Balanced accuracy is given in Equation 1.


$$\mathrm{BA}=\frac{1}{2}\;(\frac{\mathrm{TP}}{\mathrm{TP}+\mathrm{FN}}+\frac{\mathrm{TN}}{\mathrm{TN}+\mathrm{FP}})\;$$ (1)

where BA is balanced accuracy and TP, FN, TN, and FP are the counts of true positives, false negatives, true negatives, and false positives. The first bracketed term is sensitivity and the second specificity, so BA is their unweighted mean; a classifier assigning every case to the majority class attains BA = 0.500 for two classes, however extreme the class distribution. The Matthews correlation coefficient is given in Equation 2.


$$\mathrm{MCC}=\frac{\mathrm{TP}\times \mathrm{TN}-\mathrm{FP}\times \mathrm{FN}}{\sqrt{\left(\mathrm{TP}+\mathrm{FP})(\mathrm{TP}+\mathrm{FN})(\mathrm{TN}+\mathrm{FP})(\mathrm{TN}+\mathrm{FN}\right)}}\;$$ (2)

where the Matthews correlation coefficient (MCC) is the Pearson correlation between observed and predicted binary labels, ranging from −1 to +1 and taking the value 0 for a constant or random classifier. It is the strictest metric reported here because it requires above-chance performance on both classes simultaneously. Accuracy, balanced accuracy, macro *F*_1_, MCC and the area under the receiver operating characteristic (AUC-ROC) curve are reported together for every classifier.

### 2.7 Model comparison, permutation nulls and interpretability

Repeated cross-validation folds are not independent because training partitions overlap, so model comparisons use the corrected statistic of Nadeau and Bengio (2003), given in Equation 3.


$$t=\frac{\bar{d}}{\sqrt{s^{2}\;(\frac{1}{m}+\frac{n_{\text{test}}}{n_{\text{train}}})}}$$ (3)

where *d—* is the mean difference in fold-level scores between the two models compared, *s*^2^ the sample variance of those differences, *m* the number of outer folds (10 here), and *n*_test_ and *n*_train_ the sizes of a validation partition and its training partition. The ratio of partition sizes is the correction term for fold overlap; the statistic is compared against a *t*-distribution with *m* − 1 degrees of freedom. Each best model was additionally refitted on datasets with randomly permuted outcome labels, using 100 permutations for each regression target and 200 for the classification target. Hence, the smallest attainable *p*-values are 0.010 and 0.005, respectively.

Shapley Additive exPlanations (Lundberg and Lee, 2017) were computed for the best tree-based model for each outcome; the formal definition of the Shapley value and of its terms is given in the Supplementary material.

Values were computed with the tree path-dependent algorithm. Permutation importance was computed on held-out folds according to Equation 4.


$$I_{j}=s-\frac{1}{K}\sum_{k=1}^{K}s_{k,j}$$ (4)

where *I*_j_ is the importance of feature *j*, *s* the score on the untouched validation fold, *s*_k,j_ the score after the *k*th random permutation of feature *j* within that fold, and *K* the number of repeats (5 here). Rank stability was assessed by computing Equation 4 in 15 repeated folds and reporting bootstrap intervals on the resulting ranks. *Post-hoc* explanation was reported only for outcomes exceeding their permutation null, as encoded at lines 15–18 of Algorithm 1, because explaining a model that has not learned anything produces plausible narratives about associations that do not exist (Petch et al., 2022).

Heterogeneity was examined by re-estimating models within eight strata defined by age, instrumentalist status and listening quantity. Robustness was examined across eight alternative specifications, including adult-only samples, exclusion of tempo, one-hot coding of genre exposure, chained-equation imputation, complete-case analysis, alternative random seeds and models restricted to listening behavior or to musical taste alone. All analyses used Python 3.12 (Python Software Foundation, Wilmington, DE, USA) with pandas, NumPy, scikit-learn 1.8, XGBoost 3.4, SHAP 0.52, statsmodels 0.14 and Matplotlib. The alpha level was 0.05 throughout, and all tests were two-tailed. Random seeds were fixed at 42 for the primary analysis and varied across 1, 7, and 2024 in the robustness checks.

## 3 Results

### 3.1 Sample characteristics

Table 1 reports descriptive statistics. Self-reported anxiety was high on average (mean: 5.84, SD: 2.79, median: 6), consistent with a self-selected sample recruited through channels where discussion of mental health is common. Insomnia and obsessive–compulsive ratings were concentrated toward the low end, with 25% of respondents reporting the minimum value of 0 for the latter, a floor effect that restricts the variance available to be explained. Zero-order associations between listening variables and distress were uniformly small: the largest was between daily listening hours and insomnia (*r* = 0.15), followed by daily listening hours and depression (*r* = 0.14). Age correlated negatively with anxiety (*r* = −0.18) and with obsessive–compulsive ratings (*r* = −0.13). Intercorrelations among outcomes were moderate for anxiety with depression (*r* = 0.52) and weaker elsewhere, from 0.20 for depression with obsessive–compulsive ratings to 0.38 for depression with insomnia. Three-quarters of respondents (542 of 728 with valid responses, 74.5%) reported that music improves their mental health, and only 17 (2.3%) reported worsening.

### 3.2 Parametric baseline models

Table 2 reports the parametric baselines. In-sample explained variance ranged from 0.069 for obsessive–compulsive ratings to 0.128 for depression. Still, adjusted values fell to between 0.012 and 0.075, the first indication that much of the apparent fit reflects the number of parameters rather than genuine structure. Individual coefficients were mostly small, and few reached the alpha level of 0.05. Age was negatively associated with anxiety [*B* = −0.035, standard error (SE) = 0.012, *p* = 0.003] and with obsessive–compulsive ratings (*B* = −0.031, SE = 0.010, *p* = 0.002). Daily listening hours predicted insomnia (*B* = 0.161, SE = 0.051, *p* = 0.001) and obsessive–compulsive ratings (*B* = 0.106, SE = 0.047, *p* = 0.024). Metal exposure related positively to depression and insomnia, and country exposure negatively to depression. Several streaming service indicators reached the alpha level for anxiety, most plausibly as proxies for age composition rather than as platform effects.

**Table 2:** Parametric baseline models: pooled ordinary least squares regressions and binary logistic regression.

Outcome	*R* ^2^	Adj. *R*^2^	*F*	*N*	Predictors reaching *p* < 0.05 (three largest)
Anxiety	0.093	0.038	1.71	736	Service: YouTube Music (*B* = −1.418, *p* = 0.003); age (*B* = −0.035, *p* = 0.003); Service: Spotify (*B* = −1.091, *p* = 0.005)
Depression	0.128	0.075	2.64	736	Exposure: country (*B* = −0.414, *p* = 0.011); age (*B* = −0.024, *p* = 0.034); exposure: metal (*B* = 0.330, *p* = 0.036)
Insomnia	0.091	0.035	1.70	736	Daily listening hours (*B* = 0.161, *p* = 0.001); favorite genre: rap (*B* = −1.918, *p* = 0.026); exposure: metal (*B* = 0.338, *p* = 0.036)
OCD	0.069	0.012	1.45	736	Age (*B* = −0.031, *p* = 0.002); exposure: EDM (*B* = 0.365, *p* = 0.008); daily listening hours (*B* = 0.106, *p* = 0.024)
Distress	0.105	0.050	2.05	736	None at *p* < 0.05

Logistic predictor	*B*	SE	OR	95% CI	*p*
Listens while working	0.864	0.230	2.37	1.51–3.72	<0.001
Explores new music	0.566	0.216	1.76	1.15–2.69	0.009
Plays an instrument	0.507	0.245	1.66	1.03–2.68	0.038
Exposure: country	0.276	0.136	1.32	1.01–1.72	0.043
Exposure: classical	−0.244	0.123	0.78	0.62–1.00	0.047

Ordinary least squares estimates pooled across five chained-equation imputations using the rules of Rubin (1987), with HC3 standard errors; all models include the full set of 42 predictors. These are in-sample fit statistics and are not comparable with the cross-validated values in Table 3. The logistic model contrasts improvement (*n* = 542) against no effect or worsening (*n* = 186); *N* = 728, McFadden pseudo-*R*^2^ = 0.100, base rate = 0.745. OR, odds ratio; CI, confidence interval; SE, standard error.

The binary logistic model produced a McFadden pseudo *R*^2^ of 0.100 on 728 usable cases. Listening while working carried the largest association [OR = 2.37, 95% confidence interval (CI): 1.51–3.72, *p* < 0.001], corresponding to an average marginal effect of 15.1 percentage points, followed by actively exploring new music and playing an instrument. The three-category multinomial specification yielded a pseudo-*R*^2^ of 0.050, and the worsening category could not be estimated with useful precision given 17 cases.

### 3.3 Out-of-sample performance and permutation tests

Table 3, Figure 2 report nested cross-validated performance for the five regression targets. Random forests were the best-performing family for every outcome, reaching *R*^2^ of 0.068 for depression, 0.058 for the composite index, 0.032 for anxiety, 0.006 for insomnia and 0.005 for obsessive–compulsive ratings. Support vector machines performed worse than the mean-prediction baseline for four of five outcomes, reaching −0.121 for obsessive–compulsive ratings, and gradient boosting was unstable across folds.

**Table 3:** Nested cross-validated performance and permutation tests for the five regression targets.

Outcome	Model	*R* ^2^	95% CI	RMSE	*p* vs. baseline	Permutation *p*
Anxiety	Dummy (mean)	−0.004	−0.007 to −0.001	2.792		
	Elastic net	0.005	−0.012 to 0.022	2.779		
	SVM (RBF)	−0.044	−0.062 to −0.025	2.847		
	Random forest	0.032	0.017 to 0.047	2.742	0.021	0.010
	Gradient boosting (XGBoost)	0.010	−0.014 to 0.033	2.772		
Depression	Dummy (mean)	−0.005	−0.008 to −0.002	3.028		
	Elastic net	0.040	0.023 to 0.058	2.958		
	SVM (RBF)	−0.026	−0.046 to −0.006	3.058		
	Random forest	0.068	0.045 to 0.091	2.914	0.005	0.010
	Gradient boosting (XGBoost)	0.054	0.019 to 0.089	2.935		
Insomnia	Dummy (mean)	−0.019	−0.036 to −0.002	3.094		
	Elastic net	0.000	−0.030 to 0.030	3.064		
	SVM (RBF)	−0.026	−0.045 to −0.008	3.107		
	Random forest	0.006	−0.020 to 0.033	3.056	0.157	0.198
	Gradient boosting (XGBoost)	−0.016	−0.061 to 0.030	3.087		
OCD	Dummy (mean)	−0.011	−0.018 to −0.004	2.841		
	Elastic net	0.003	−0.005 to 0.012	2.821		
	SVM (RBF)	−0.121	−0.157 to −0.084	2.992		
	Random forest	0.005	−0.003 to 0.014	2.818	0.028	0.040
	Gradient boosting (XGBoost)	−0.034	−0.051 to −0.018	2.873		
Distress	Dummy (mean)	−0.007	−0.012 to −0.002	0.704		
	Elastic net	0.018	−0.001 to 0.036	0.695		
	SVM (RBF)	0.004	−0.004 to 0.012	0.700		
	Random forest	0.058	0.044 to 0.071	0.681	<0.001	0.010
	Gradient boosting (XGBoost)	0.037	0.012 to 0.062	0.688		

Ten outer folds from five-fold cross-validation repeated twice, with hyperparameter selection nested inside three-fold inner loops; these are the primary performance estimates of the study. Confidence intervals are normal approximations around the mean of the outer fold scores. The *p* vs. baseline column gives the corrected *t*-test of Equation 3 comparing random forests against the mean-prediction baseline. Permutation *p*-values are from 100 label permutations, yielding a minimum attainable value of 0.010. The composite distress index is standardized, so its RMSE is on a different scale from the 0 to 10 ratings. It is reported as a secondary summary measure rather than as a primary outcome, for the reasons given in Section 2.2. SVM (RBF), Support Vector Machine; RMSE, root mean squared error; CI, confidence interval.

**Figure 2:**
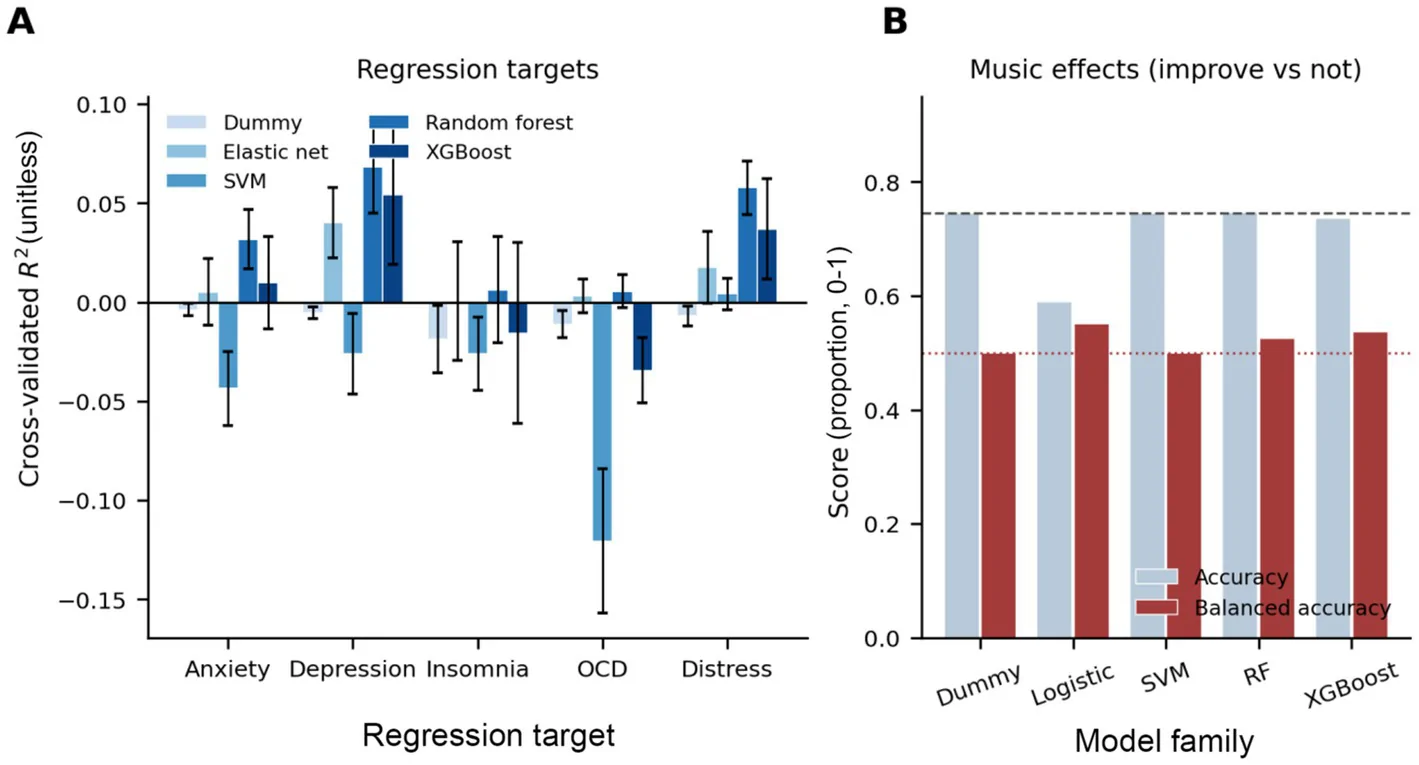
Out-of-sample performance against trivial baselines. **(A)** Cross-validated *R*^2^ for the five regression targets across five model families, with 95% confidence intervals from ten outer folds; the dummy regressor predicts the training mean. **(B)** Accuracy (light bars) and balanced accuracy (dark bars) for the self-attributed effect of music, with majority-class accuracy shown as a dashed line and chance-level balanced accuracy as a dotted line. The divergence between the two metrics in panel B is the central result of the study.

Corrected comparisons using Equation 3 showed that random forests exceeded the mean-prediction baseline for the composite index (*t* = 5.92, *p* < 0.001), depression (*t* = 3.64, *p* = 0.005), anxiety (*t* = 2.79, *p* = 0.021) and obsessive–compulsive ratings (*t* = 2.62, *p* = 0.028), but not insomnia (*t* = 1.54, *p* = 0.157). Differences between random forests and the second-best learner were not statistically significant for any outcome (*p* = 0.065 to 0.611). Hypothesis H2 is therefore supported in both parts: explanatory power fell far below the 0.15 threshold, and the non-linear ensemble did not reliably beat the regularized linear model.

Permutation results appear in the final column of Table 3 and in Figure 3. Under randomly permuted labels, cross-validated *R*^2^ centered on negative values between −0.034 and −0.016, the expected behavior of a flexible model fitted to noise. Observed values for depression, the composite index and anxiety fell far outside the null at the smallest attainable value (*p* = 0.010), and obsessive–compulsive ratings cleared it marginally (*p* = 0.040). Insomnia did not (*p* = 0.198), so no interpretability analysis is reported for that outcome.

**Figure 3:**
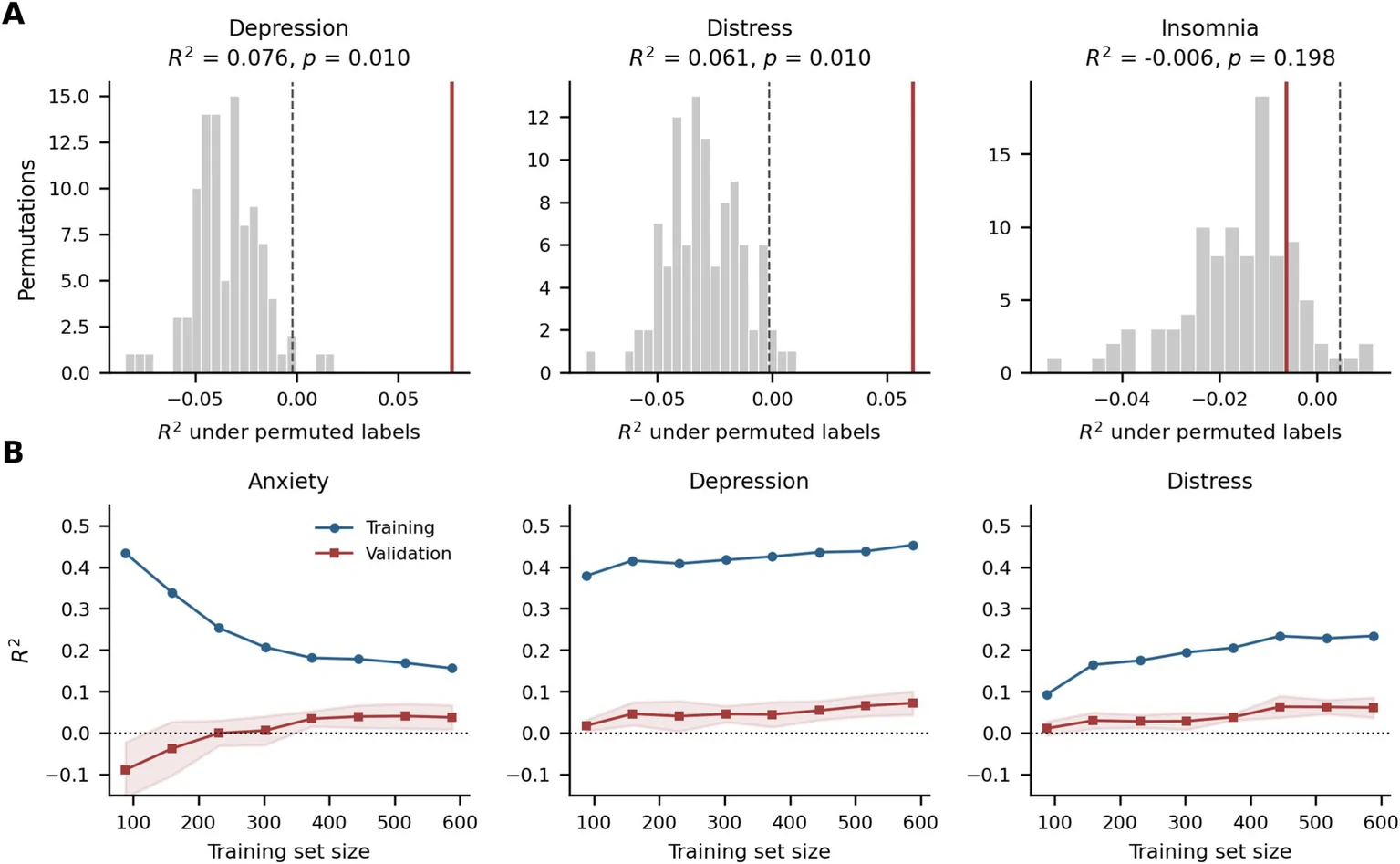
Signal detection and sample-size diagnostics. **(A)** Permutation null distributions for three representative outcomes; the solid vertical line marks the observed score and the dashed line the 97.5th percentile of the null. Depression and the composite distress index fall far outside their nulls, whereas insomnia does not. **(B)** Learning curves for three outcomes, showing training performance (upper line) and validation performance (lower line, with shaded standard deviation) across eight training set sizes. Validation performance plateaus near 400 respondents while the gap to training performance persists, indicating a ceiling imposed by the informativeness of the variables rather than by sample size.

### 3.4 Classification of the self-attributed effect of music

Table 4 reports classification performance, and these results constitute the clearest finding of the study. The majority-class classifier achieves 74.5% accuracy by predicting improvement for every respondent. The best-performing learner, a random forest, achieves 74.7%. The difference of 0.2 percentage points is not statistically significant (*t* = 0.13, *p* = 0.896). Every metric sensitive to class imbalance diverges from accuracy: balanced accuracy peaked at 0.551, the Matthews correlation coefficient at 0.135, and nested AUC at 0.578, which did not clear the baseline after correction (*t* = 2.18, *p* = 0.057). Hypothesis H4 is supported without qualification.

**Table 4:** Nested cross-validated classification performance for the self-attributed effect of music.

Specification	Model	Accuracy	Balanced accuracy	Macro F1	MCC	AUC
Binary	Dummy (majority)	0.745	0.500	0.427	0.000	0.500
	Logistic (elastic net)	0.589	0.551	0.488	0.090	0.576
	SVM (RBF)	0.745	0.500	0.427	0.000	0.578
	Random forest	0.747	0.525	0.489	0.135	0.562
	Gradient boosting (XGBoost)	0.736	0.537	0.520	0.118	0.551
Three-class	Dummy (majority)	0.745	0.333	0.285	0.000	–
	Logistic (elastic net)	0.479	0.338	0.312	0.032	–
	SVM (RBF)	0.589	0.333	0.236	0.000	–
	Random forest	0.742	0.336	0.293	0.013	–
	Gradient boosting (XGBoost)	0.747	0.342	0.304	0.074	–

*N* = 728. Base rate for improvement is 0.745 in the binary specification; chance-level balanced accuracy is 0.500 for two classes and 0.333 for three. The worsening category (*n* = 17) is not estimable, and AUC is not defined for the three-class specification. Note the divergence between accuracy and every imbalance-sensitive metric. MCC, Matthews correlation coefficient; AUC-ROC, area under the receiver operating characteristic curve.

The behavior of the tuned support vector machine illustrates the mechanism directly. Hyperparameter search selected a heavily regularized configuration (*C* = 0.077, gamma = 0.0014), and at the standard decision threshold this model assigned the improvement label to all 728 respondents in every fold. Its confusion matrix contains a column of zeros and its balanced accuracy is exactly 0.500. It is, in operational terms, a constant function, yet it attains the accuracy figure that would be read as evidence that effects of music on mental health are predictable.

A calibration failure encountered during this analysis is reported here because it is easily reproduced inadvertently. The decision function of that model ranked cases with an AUC of 0.619, but the Platt-scaled probabilities derived from it (Platt, 1999), pooled across folds, ranked cases at 0.381. The model had not reversed its predictions. The sigmoid fitted to a nearly flat decision surface varied in slope and sign across folds, so probabilities that were internally consistent within a fold were not comparable between folds, and pooling destroyed the ranking. This is a failure of probability calibration under a degenerate decision surface, not a failure of discrimination.

Calibration and confusion diagnostics were therefore computed from the elastic net logistic model, whose probability estimates are comparable across folds, and appear in Figure 4. Tuned once on the full sample and evaluated out of fold, that model achieved accuracy 0.621, balanced accuracy 0.590, AUC 0.622, sensitivity 0.653 and specificity 0.527. These figures exceed the nested estimates for the same family in Table 4 because hyperparameters were selected using the whole sample rather than within each training fold; they characterize the shape of the errors and the quality of the probabilities, whereas Table 4 reports unbiased estimates. The Brier score of 0.237 is worse than the 0.190 obtained by predicting the base rate for everyone, indicating that the modest gain in discrimination was purchased at a cost of calibration. The three-category specification performed no better, achieving a balanced accuracy of 0.342 against a chance floor of 0.333 (*p* = 0.514). With 17 respondents in the worsening category, that category is not estimable.

**Figure 4:**
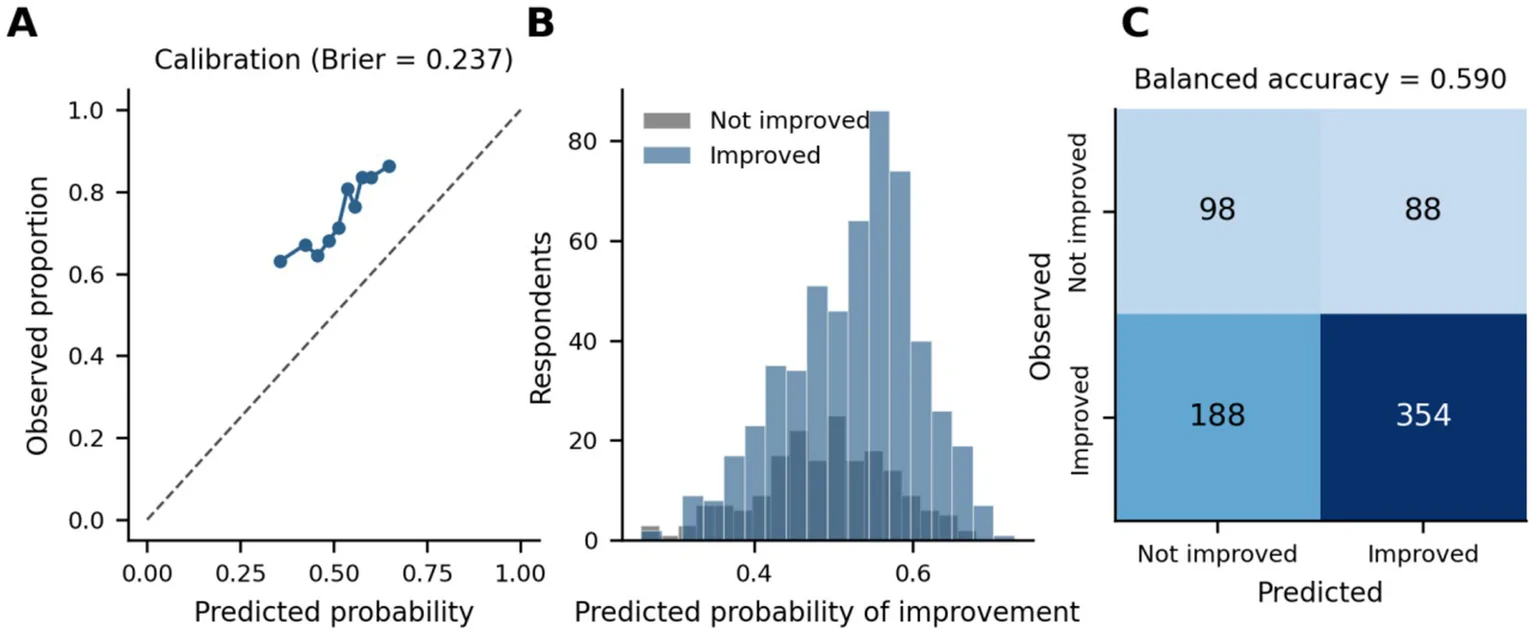
Classification diagnostics for the self-attributed effect of music, computed from the elastic net logistic model using out-of-fold predictions. **(A)** Calibration curve with the diagonal indicating perfect calibration; the Brier score of 0.237 is worse than the 0.190 obtained by predicting the base rate for every respondent. **(B)** Distribution of predicted probabilities separately for respondents who reported improvement and those who did not, showing substantial overlap. **(C)** Out-of-fold confusion matrix, with balanced accuracy of 0.590.

### 3.5 Feature importance and functional form

Following the gate at lines 15–18 of Algorithm 1, interpretability analyses were run only for the four outcomes, clearing their permutation null. Selected hyperparameters were themselves informative: the chosen forests were heavily constrained, with maximum depth of 3 for anxiety, a maximum feature fraction of 0.2 for depression, and a minimum of 20 observations per leaf for the composite index, the expected outcome when little structure is available to fit.

Table 5 reports feature importance. For depression, mean absolute SHAP values ranked metal exposure first (0.247) and age second (0.199), with rock, rap, daily listening hours, and hip hop close behind. Permutation importance from Equation 4 gave a partially different ordering, placing age first (0.028) and metal second (0.021), and the bootstrap rank intervals explain the disagreement: age has a median rank of 1 but an interval extending to 26, while metal has a median rank of 2 with an interval reaching 9. Only metal and hip hop exposure had permutation importance intervals excluding zero. Feature rankings on this dataset are unstable, and a SHAP ordering derived from them should not be treated as a settled finding. For anxiety, age dominated (SHAP: 0.286, permutation importance: 0.075), an order of magnitude above the next predictor. Hypothesis H3 is supported: age is among the strongest predictors throughout and is plausibly upstream of apparent genre effects, since preference for metal and rap concentrates in younger respondents who also report higher distress. SHAP interaction values placed age and metal exposure as the strongest interacting pair.

**Table 5:** Feature importance for the two best-predicted individual outcomes.

Outcome	Rank	Feature	Mean |SHAP|	Permutation importance	95% CI	Median rank (95% CI)
Depression	1	Exposure: metal	0.247	0.0211	0.0027 to 0.0404	2 (1–9)
	2	Age	0.199	0.0279	−0.0012 to 0.0506	1 (1–26)
	3	Exposure: rock	0.177	0.0123	−0.0025 to 0.0298	4 (2–36)
	4	Exposure: rap	0.149	0.0101	−0.0078 to 0.0245	4 (1–40)
	5	Daily listening hours	0.144	0.0073	−0.0074 to 0.0216	6 (2–39)
	6	Exposure: hip hop	0.119	0.0111	0.0008 to 0.0245	4 (3–22)
Anxiety	1	Age	0.286	0.0745	0.0098 to 0.1130	1 (1–4)
	2	Exposure: folk	0.121	0.0060	−0.0037 to 0.0114	2 (2–28)
	3	Exposure: rock	0.055	0.0015	−0.0024 to 0.0061	11 (2–41)
	4	Exposure: metal	0.042	0.0023	−0.0014 to 0.0057	4 (3–32)
	5	Daily listening hours	0.041	−0.0005	−0.0071 to 0.0026	18 (4–42)
	6	Exposure: pop	0.040	0.0028	−0.0043 to 0.0113	5 (1–42)

Ranks are by mean absolute SHAP value. Permutation importance was computed on held-out folds across 15 repeated partitions, with bootstrap percentile intervals; intervals including zero indicate features whose contribution is not distinguishable from noise. The corresponding table for the composite distress index is provided in the Supplementary material. SHAP, Shapley additive explanations; CI, confidence interval.

Figure 5 displays the SHAP summary for depression and Figure 6 the dependence structure. The relationship between daily listening hours and predicted depression is not monotonic: contributions are near zero from roughly 2–5 h and rise sharply above approximately 6 h, so the association is concentrated in the upper tail of listening quantity rather than being distributed across it. Metal exposure shows a step rather than a slope, shifting at the boundary between rarely and sometimes. Hypothesis H5 is therefore supported.

**Figure 5:**
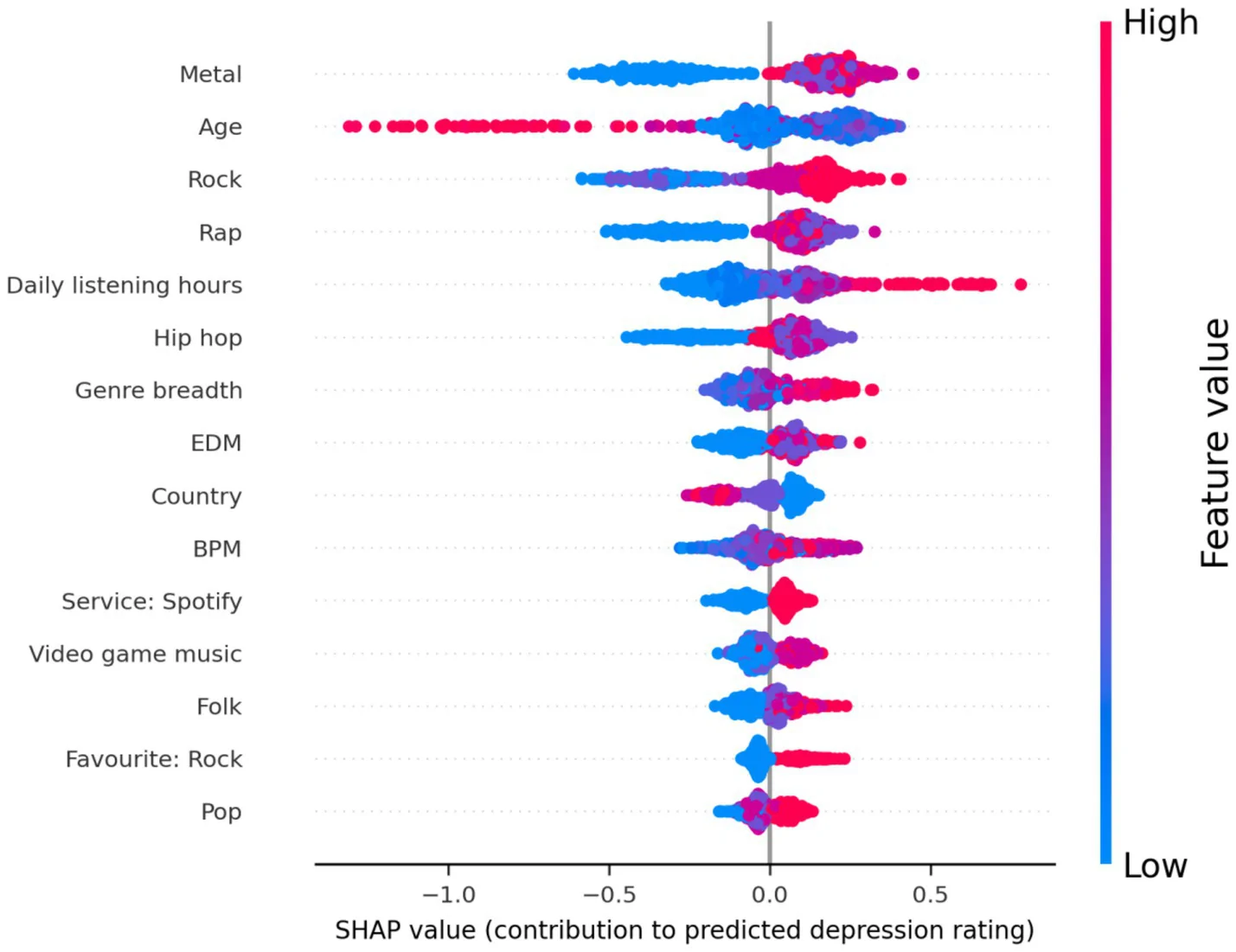
SHAP summary for the depression model, showing the 15 highest-ranked features. Each point represents one respondent; horizontal position gives the contribution of that feature to the predicted depression rating and color gives the feature value, from low (light) to high (dark). Metal exposure and age carry the widest contributions, but the horizontal spread of all features is narrow, consistent with the small overall explanatory power reported in Table 3.

**Figure 6:**
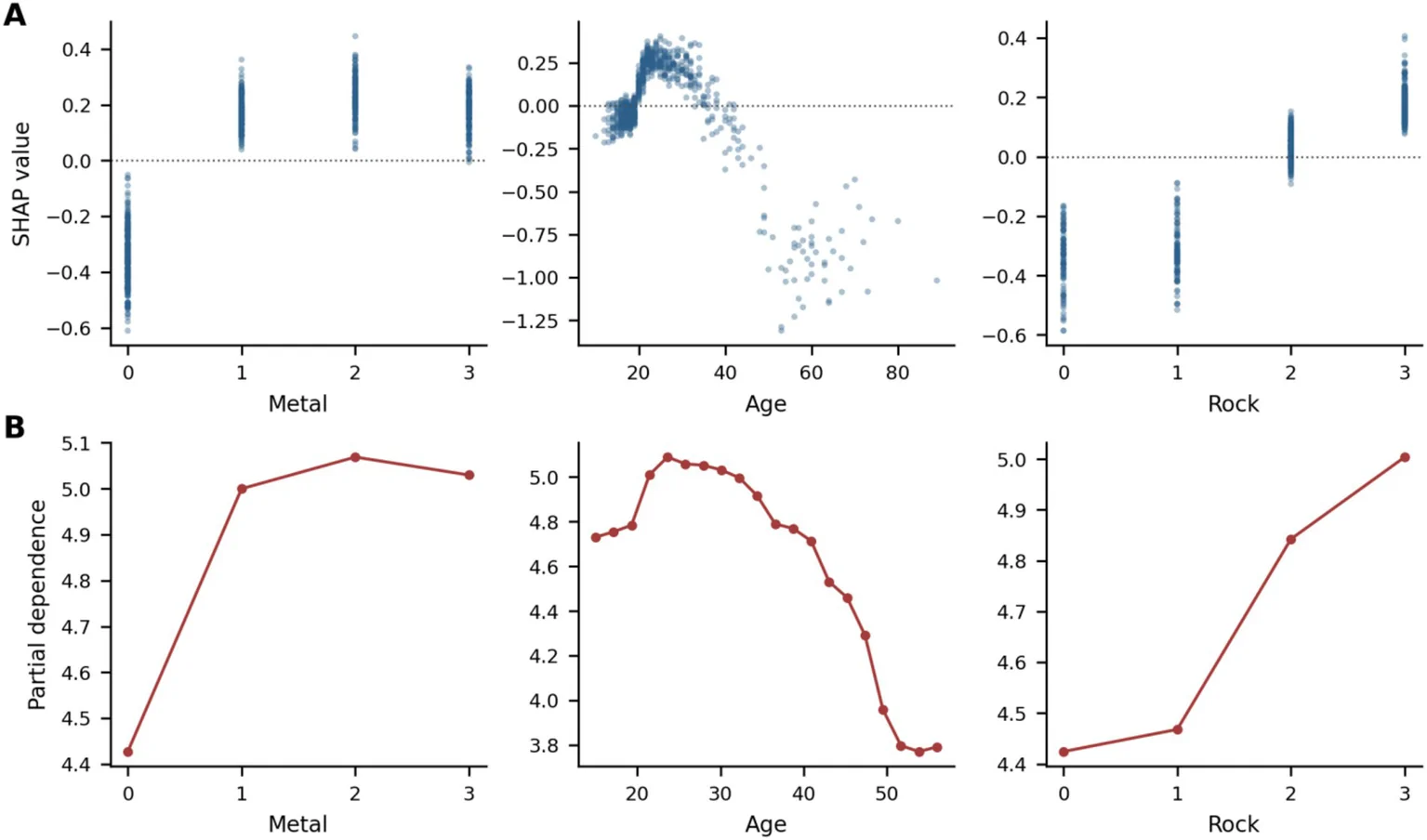
Functional form of the three highest-ranked predictors of depression. **(A)** SHAP dependence plots, with each point one respondent. **(B)** Partial dependence plots for the same three predictors. The relation between daily listening hours and predicted depression is non-monotonic, remaining near zero from approximately 2–5 h and rising above approximately 6 h; metal exposure shows a step at the boundary between rarely and sometimes rather than a graded slope.

### 3.6 Heterogeneity and robustness

Subgroup analyses showed substantial variation, the majority of it attributable to sample size rather than genuine moderation. Among respondents aged 18 and over, depression reached *R*^2^ of 0.077 and the composite index 0.069. Among respondents under 18, both fell below zero (−0.037 and −0.280), indicating that the models performed worse than the subgroup mean. With 153 respondents and a restricted age range within that stratum, this is expected and should not be read as evidence that adolescent distress is unpredictable in principle. Instrumentalists showed somewhat higher explained variance for depression than non-instrumentalists (0.096 vs. 0.055), with substantially overlapping intervals.

Table 6 reports robustness. Because the purpose is to compare specifications rather than to estimate performance, every row uses a single 5-fold cross-validation with hyperparameters selected on the full sample, which is why the reference row differs slightly from the nested estimates in Table 3 (depression 0.076 vs. 0.068; anxiety 0.037 vs. 0.032; composite 0.061 vs. 0.058). The direction of the gap is expected, since tuning on the full sample leaks a small amount of information into the score. Read as a comparison between rows, the conclusions are stable: excluding tempo, one-hot coding of genre exposure and chained-equation imputation all left the results materially unchanged, and across three alternative seed sets the reference estimates varied by less than 0.015.

**Table 6:** Robustness of cross-validated *R*^2^ across alternative specifications.

Specification	*n*	Anxiety	Depression	Insomnia	OCD	Distress
Reference (single five-fold)	736	0.037	0.076	−0.006	0.004	0.061
Adults only (18+)	582	0.026	0.077	0.030	−0.009	0.069
Genre exposure one-hot coded	736	0.041	0.063	−0.013	0.001	0.066
Chained-equation imputation	736	0.037	0.069	−0.007	0.005	0.060
Complete cases only	610	0.047	0.058	0.003	−0.001	0.051
Alternative seeds (mean of 3)	736	0.039	0.064	0.019	0.001	0.062
Listening behavior only	736	0.026	0.033	−0.011	0.001	0.058
Musical taste only	736	−0.001	0.056	−0.019	−0.017	0.017

All rows use a single five-fold cross-validation with hyperparameters selected for the reference specification, so rows are comparable with one another but not with the nested estimates in Table 3. Listening behavior excludes only genre exposure and favorite genre; musical taste excludes only age, listening hours, tempo, and engagement indicators. Negative values indicate performance worse than predicting the sample mean. OCD, obsessive–compulsive rating.

The two final rows test H1 directly. Restricting the model to listening behavior yielded *R*^2^ of 0.026 for anxiety and 0.033 for depression, while restricting it to musical taste yielded −0.001 for anxiety and 0.056 for depression. For anxiety and for the composite index, the hypothesis holds, with taste alone predicting essentially nothing and behavior carrying what little signal exists. For depression, the reverse holds, with genre exposure retaining the majority of the predictive content when behavioral variables are removed. H1 is therefore partially supported.

## 4 Discussion

This study examined how much musical preferences and listening behaviors can reveal to an algorithm about an individual’s self-reported psychological distress. The answer, on these data and with this measurement, is a small amount for depression and general distress, less for anxiety, a marginal amount for obsessive–compulsive ratings, and nothing distinguishable from chance for insomnia. The upper bound is a cross-validated *R*^2^ of approximately 0.07.

H1 anticipated that listening behavior would outperform taste, on the reasoning that theory locates the psychologically active variable in how music is used rather than in what is played (Saarikallio et al., 2015; Kanagala et al., 2021). The results split. For anxiety and general distress, the hypothesis held, with taste alone explaining essentially nothing. For depression, it failed, with genre exposure retaining the majority of the predictive content when behavioral variables were removed. The most parsimonious reading is that metal and rock exposure function partly as age proxies and partly as markers of a listening style this dataset cannot observe directly, a reading supported by the age-by-metal interaction. This carries a constructive implication for measurement: a dataset recording a validated measure of adaptive and maladaptive engagement alongside genre exposure would plausibly perform better, since correlations near 0.39 between maladaptive engagement and depression totals have been reported (Kanagala et al., 2021), substantially stronger than anything observable in these variables. The ceiling documented here is a ceiling for this measurement, not for the construct.

The clearest result is also the one that depends least on theory. Three-quarters of respondents said that music improves their mental health. A classifier that assigns that response to every respondent therefore achieves 74.5% accuracy, and no model in this study meaningfully improved on it. The best-performing model at the standard threshold made an identical prediction for all 728 respondents. Reporting accuracy without balanced accuracy would yield a figure that describes only the marginal distribution of the outcome.

This matters beyond the present dataset. Tools claiming to infer psychological state from listening behavior are evaluated, when evaluated publicly at all, on metrics of exactly this kind. The correction is computationally trivial and can be automated. We would encourage researchers, editors, and reviewers who assess their study to require that any classification result for an imbalanced outcome be accompanied by the corresponding majority-class baseline and at least one imbalance-sensitive metric, so the comparison is visible at the point of reading. The calibration failure described in Section 3.4 reinforces the point: when a decision surface is nearly flat, quantities usually dependable stop being dependable, and the failure is silent rather than obvious.

The learning curves in Figure 3B distinguish two explanations that are often conflated. If the constraint were sample size, validation performance would still be climbing at the largest training partitions. It does not. Validation performance flattens by roughly 400 respondents for anxiety and rises only marginally thereafter for depression, while training performance remains far higher throughout. The models are not limited by the quantity of available data; they are extracting nearly everything these variables contain, and that amount is small. This places the study alongside a broader literature on the limits of prediction for psychological outcomes, in which reported accuracy declines as sample sizes grow, suggesting that many small-sample results were never real (Flint et al., 2021).

The methodological message extends beyond music psychology to the wider digital mental health biomarker literature. Signals as varied as smartphone mobility, phone and call use, sleep and vocal characteristics are now cataloged as candidate markers of psychiatric state (Bufano et al., 2023). The evaluation problem they raise is the one this study illustrates: moderate accuracy reported from an intuitive behavioral or acoustic signal, obtained on a small sample with weak labels, little or no external validation, and no trivial baseline against which the figure can be read. Where that has been assessed, the shortfall is systematically measurable. A scoping review of voice-based screening for depression and anxiety found a median total sample size of 118 participants; external validation was absent in 68% of evaluations. Only 11% of evaluations were rated as having high clinical readiness, and accuracy was reported without the accompanying class distribution, a specific barrier to interpretation (Muntean-Codrea et al., 2026). Vilenchik et al. (2025) make the complementary point from the design side, showing in voice-based depression detection that how the behavioral sample is collected, including how short recordings are elicited and how labels and class balance are constructed, drives measured performance rather than model architecture alone, and reporting their result as a lift over an explicit balanced baseline rather than as an unreferenced accuracy figure. Their conclusion and ours converge from opposite directions: their framework improves what the data contain, while the protocol used here constrains what may be claimed from data already collected. Both are needed before a brief, low-burden sample of behavior can be described as a screening instrument.

A distinction between classes of signal follows from this, and it bounds the generality of our conclusion. Music listening records are an indirect behavioral trace. What a person plays is the joint outcome of taste, availability, social setting, platform recommendations, and habit, and, in theory, the psychologically active variable is not the track but the function that listening serves (Schäfer et al., 2013; Saarikallio et al., 2015; Kanagala et al., 2021), which these variables do not capture. Voice, by contrast, is a produced physiological signal that carries information about respiration, phonation, articulation rate, psychomotor state, and autonomic regulation, and stands in a more proximal relation to affect; the same applies to other physiological phenotypes such as actigraphy or heart rate variability. We therefore do not conclude that digital mental health signals are weak in general. The conclusion is narrower: an intuitive behavioral proxy is a hypothesis about information content rather than a biomarker, and it should be required to clear a trivial baseline and a permutation null under nested validation before it is treated as one. The protocol applied here is computationally inexpensive and indifferent to the modality of the signal, so it transfers without modification to voice, movement, typing, or listening data, and that is where we would expect it to be useful for the future work on low-burden screening.

A low ceiling has two possible sources with different implications: the predictors may be uninformative, or the outcome may be measured too unreliably for any predictor to track it. Under classical test theory, these can be separated arithmetically. If the outcome has reliability r and the predictors are measured without error, the observed squared multiple correlation is attenuated by that reliability, so the disattenuated value is the observed value divided by r. The maximum attainable observed *R*^2^ given perfect predictors equals r itself.

For the composite index, which is reported here as a secondary summary measure rather than a primary outcome, its reliability is estimable from these data; *that estimability is the main reason it is retained*. With a Cronbach alpha of 0.659, the observed cross-validated *R*^2^ of 0.058 corresponds to a disattenuated value of 0.088, so roughly a third of the shortfall is attributable to unreliability in the outcome rather than to the music variables. For the four single items, no reliability estimate is available, since each was administered once with no test–retest interval. Under assumed reliabilities of 0.60, 0.70, and 0.80, spanning the range typically reported for single-item distress ratings, the observed depression value of 0.068 corresponds to 0.114, 0.098, and 0.085, and the anxiety value of 0.032 corresponds to 0.053, 0.045, and 0.040. Measurement error is therefore a real and quantifiable component of the ceiling, and the low values reported here should not be attributed solely to the music variables. However, correcting for it does not change the substantive picture: even under the most favorable assumption entertained, the disattenuated ceiling remains below 0.12, which supports population-level description rather than individual-level inference.

The gating rule adopted here speaks to a wider problem in applied explainable machine learning. Attribution methods return an answer whenever they are called. They do not report that the model being explained performs no better than its own null model, and a SHAP summary produced from such a model looks indistinguishable from one produced by a model that has learned something. Explanation can therefore lend the appearance of mechanism to a fitted pattern that is noise, a hazard raised previously for clinical prediction (Petch et al., 2022). The present analysis gives a concrete instance: had the gate not been applied, insomnia would have yielded a complete and entirely presentable set of feature attributions from a model that does not beat chance. Reporting attributions only after a model clears an explicit null is a low-cost and generally applicable safeguard.

One caution applies to every number reported above. The MxMH file is a single archived dataset that has been analyzed a great many times publicly, in teaching exercises, competition notebooks, and published studies, and the analytic choices that circulate such a file are shaped by results already seen on it. This is collective overfitting, and no internal validation procedure can detect it because nested cross-validation, permutation nulls, and corrected comparisons all operate within the same archive. The present study is not exempt. The direction of the findings is comparatively robust to this concern, since it is difficult to overfit a reused dataset toward a negative result. Still, the specific effect sizes are not: the cross-validated *R*^2^ of 0.068 for depression should be read as an estimate from one heavily reused convenience sample rather than as a population quantity. Independent replication on a separately collected sample, ideally one that also records the functions music serves alongside genre exposure, is required before any specific value reported here is treated as settled.

Three implications follow. For digital mental health applications, musical preference is not, on this evidence, a usable individual-level marker of psychological state; an *R*^2^ of 0.07 supports population-level description, and a system acting on such a signal would be wrong far more often than it would be right. For music recommendation, the finding is more encouraging in one respect, since taste carries so little information about distress that the privacy risk of inferring mental health from listening histories is, with variables of this kind, smaller than sometimes feared. For research practice, the recommendation is concrete: report a trivial baseline, report a permutation null, nest hyperparameter selection and gate *post-hoc* interpretation on the model having beaten its own null.

## 5 Limitations

The design is cross-sectional and observational, so no causal or directional claim is supported; nothing in these results speaks to whether music affects mental health. The sample is a self-selected online convenience sample with a median age of 21 and no measurement of gender, geography, education, income or clinical history, and self-selection into a survey about Music and Mental Health plausibly over-represents the belief that music helps, consistent with the observed 74.5% endorsement rate. Generalization to any defined population is not warranted, and the sample cannot be weighted or post-stratified toward one, because the variables needed to do so were never collected. The outcome measures are single self-anchored items with no validated instrument and no clinical corroboration; their consequences are quantified in Section 4. The obsessive–compulsive item is the most doubtful, since colloquial usage diverges sharply from the clinical construct. Self-reported tempo and listening hours carry estimation error, evidenced directly by impossible values in the archived file. The self-attributed effect of music is a belief rather than an observed change, and its imbalance leaves the worsening category inestimable at 17 respondents. The presence of 153 respondents under 18 in a public file without documentation of parental consent constrains interpretation, so an adult-only sensitivity analysis is reported throughout and no conclusion specific to minors is drawn. Finally, this dataset has been analyzed many times in public, so collective overfitting to a single archived file is a real risk from which the present study is not immune; independent replication on a separate sample would be required before these estimates were treated as settled.

## 6 Conclusion

Musical preference and listening behavior carry a small but genuine amount of predictive information about self-reported psychological distress. The ceiling is a cross-validated *R*^2^ of approximately 0.07 for depression and general distress, *the latter a secondary composite measure of modest internal consistency,* lower for anxiety and obsessive–compulsive ratings, and indistinguishable from chance for insomnia. *These values are estimates from a single self-selected online convenience sample with unvalidated single-item outcomes and no demographic weighting variables, drawn from an archive that has been analyzed publicly many times; they describe this dataset rather than any defined population, and no specific effect size reported here should be treated as settled in the absence of independent replication.* Correcting for the unreliability of the outcome measures raises that ceiling but does not change its order of magnitude. A high accuracy figure for the categorical outcome in this dataset reflects the base rate of an imbalanced variable rather than discriminative skill, demonstrated here by a model that achieves 74.7% accuracy while making identical predictions for every respondent. The methodological lesson generalizes beyond this dataset, *and past music*, *to any low-burden behavioral or physiological sample proposed as a digital marker of mental health*: where an intuitive theory predicts that a signal should exist, flexible models and permissive validation will reliably produce numbers that appear to confirm it. Trivial baselines, permutation nulls, nested validation and interpretation gated on those checks separate a predictive finding from a plausible-looking artifact.

## Data Availability

The dataset analysed in this study is publicly available through Kaggle (Rasgaitis, 2022). The archived file used here contains 736 records and 33 variables, with submission timestamps from 27 August 2022 to 9 November 2022. The complete analysis code, including the preprocessing pipeline, the nested cross-validation procedure, the permutation tests and the scripts generating every table and figure reported here, is available from the corresponding author on reasonable request and will be deposited in a public archive on acceptance.
