# Supplementary material for: How much can music listening habits tell us about mental health? An interpretable machine learning benchmark using genre preference and self-reported anxiety, depression, insomnia, and obsessive–compulsive symptoms
Source: Front Psychol. 2026 Oct 26;17:1970053. doi: 10.3389/fpsyg.2026.1970053 (PMC13746165; doi:10.3389/fpsyg.2026.1970053)
Supplement: Supplementary file 1 [file supplementary_file_2.docx]

**Supplementary Material**

How Much Can Music Listening Habits Tell Us About Mental Health? An Interpretable Machine Learning Benchmark of Genre Preference and Self-Reported Anxiety, Depression, Insomnia and Obsessive-Compulsive Symptoms

**S1. Model specifications and definitions of terms**

This section holds the four standard specifications that were presented in the main text at first submission and were moved here during revision, at the request of a reviewer, to reduce the technical density of the Methods. Nothing has been altered: each equation and each definition of terms is reproduced exactly as it was submitted. Equations retained in the main text are numbered 1 to 4 there; the equations below are numbered S1 to S4 to keep the two sequences distinct.

**S1.1 Ordinary least squares regression (Section 2.4)**

Ordinary least squares models were estimated for each outcome with heteroskedasticity-consistent HC3 standard errors and all 42 predictors entered simultaneously, as given in Eqn. S1.

$$y_{i} = \beta_{0} + \sum_{j=1}^{p} \beta_{j}x_{ij} + \varepsilon_{i}$$

(S1)

where *y*_i_ is the distress rating of respondent *i* on the 0 to 10 scale, *x*_ij_ is the value of predictor *j* for that respondent, *β*_0_ is the intercept, *β*_j_ is the partial regression coefficient for predictor *j*, *p* is the number of predictors (42 in every model) and *ε*_i_ is a respondent-specific error term.

**S1.2 Binary logistic regression for the self-attributed effect of music (Section 2.4)**

For the self-attributed effect of music, a binary logistic model contrasting improvement against all other responses was estimated as in Eqn. S2.

$$ln\left( \frac{\pi_{i}}{1 - \pi_{i}} \right) = \beta_{0} + \sum_{j=1}^{p} \beta_{j}x_{ij}$$

(S2)

where *π*_i_ is the probability that respondent *i* reports improvement, so the left-hand side is the log odds of that response. Exponentiating *β*_j_ gives the odds ratio.

**S1.3 Gradient boosting objective (Section 2.5)**

The regularised objective minimised by the gradient boosted trees at each stage is given in Eqn. S3.

$$L = \sum_{i=1}^{n} l\left( y_{i}, ŷ_{i} \right) + \sum_{k=1}^{K} \left[ \gamma T_{k} + \frac{1}{2} \lambda{\|w_{k}\|}^{2} \right]$$

(S3)

where *L* is the total objective, *n* the number of training observations, *l* a differentiable convex loss between the observed value *y*_i_ and the prediction *ŷ*_i_, *K* the number of trees, *T*_k_ the number of leaves in tree *k*, ‖*w*_k_‖^2^ the squared Euclidean norm of the leaf-weight vector, *γ* the per-leaf complexity penalty and *λ* the penalty on squared leaf weights.

**S1.4 Shapley value (Section 2.7)**

The Shapley value of feature j for a single observation is defined in Eqn. S4. Values were computed with the tree path-dependent algorithm.

$$\varphi_{j}\left( x \right) = \sum_{S \subseteq F\setminus\{j\}} \frac{|S|! \left( |F| - |S| - 1 \right)!}{|F|!} \left[ f_{S\cup\{j\}}\left( x_{S\cup\{j\}} \right) - f_{S}\left( x_{S} \right) \right]$$

(S4)

where *φ*_j_(*x*) is the contribution of feature *j* to the prediction for observation *x*, *F* the full feature set, *S* any subset excluding *j*, |·| set cardinality and the backslash set difference. The term *f*_S_ is the model prediction using only the features in *S*. The leading fraction is the combinatorial weight assigned to each subset, ensuring that all feature contributions sum to the difference between the prediction for *x* and the average prediction.

Note. Quantile regression results at the 25th, 50th and 75th percentiles, the multinomial logistic output, average marginal effects, final hyperparameter settings, the full genre exposure frequency table, the correlation matrix, variance inflation factors, the complete coefficient tables, the feature importance table for the composite distress index, SHAP interaction values, the full subgroup analysis and the complete robustness matrix are provided in the remaining sections of the Supplementary Material.
